# “Always Look on the Bright Side of Life!” – Higher Hypomania Scores Are Associated with Higher Mental Toughness, Increased Physical Activity, and Lower Symptoms of Depression and Lower Sleep Complaints

**DOI:** 10.3389/fpsyg.2017.02130

**Published:** 2017-12-12

**Authors:** Leila Jahangard, Anahita Rahmani, Mohammad Haghighi, Mohammad Ahmadpanah, Dena Sadeghi Bahmani, Ali R. Soltanian, Shahriar Shirzadi, Hafez Bajoghli, Markus Gerber, Edith Holsboer-Trachsler, Serge Brand

**Affiliations:** ^1^Research Center for Behavioral Disorders and Substances Abuse, Hamadan University of Medical Sciences, Hamadan, Iran; ^2^Center for Affective, Stress and Sleep Disorders (ZASS), Psychiatric Clinics (UPK), University of Basel, Basel, Switzerland; ^3^Department of Biostatistics, School of Public Health, Hamadan University of Medical Sciences, Hamadan, Iran; ^4^Iranian National Center for Addiction Studies, Tehran University of Medical Sciences, Tehran, Iran; ^5^Division of Sport and Psychosocial Health, Department of Sport, Exercise and Health, Faculty of Medicine, University of Basel, Basel, Switzerland; ^6^Substance Abuse Prevention Research Center, Department of Psychiatry, Kermanshah University of Medical Sciences, Kermanshah, Iran; ^7^Sleep Disorder Research Center, Department of Psychiatry, Kermanshah University of Medical Sciences, Kermanshah, Iran

**Keywords:** hypomania, depression, sleep, mental toughness, physical activity

## Abstract

**Background:** In the present study, we explored the associations between hypomania, symptoms of depression, sleep complaints, physical activity and mental toughness. The latter construct has gained interest for its association with a broad variety of favorable behavior in both clinical and non-clinical samples.

**Subjects and Methods:** The non-clinical sample consisted of 206 young adults (*M* = 21.3 years; age range: 18–24 years; 57.3% males). They completed questionnaires covering hypomania, mental toughness, symptoms of depression, physical activity, and sleep quality.

**Results:** Higher hypomania scores were associated with higher mental toughness, increased physical activity, lower symptoms of depression and lower sleep complaints. No gender differences were observed. Higher hypomania scores were predicted by higher scores of mental toughness subscales of control and challenge, and physical activity.

**Conclusion:** The pattern of results suggests that among a non-clinical sample of young adults, self-rated hypomania scores were associated with higher scores on mental toughness and physical activity, along with lower depression and sleep complaints. The pattern of results further suggests that hypomania traits are associated with a broad range of favorable psychological, behavioral and sleep-related traits, at least among a non-clinical sample of young adults.

## Introduction

For at least three reasons there is growing interest in the mood state of hypomania: First, hypomanic states and bipolar disorders may be underdiagnosed (Angst et al., 2003, 2010): As data from epidemiologic studies suggest, bipolar disorders, including hypomania, might be as frequent as unipolar depressive disorders (Merikangas et al., 2007). Second, instead of clear-cut categories between healthy and inconspicuous mood states and ‘psychopathology,’ research focuses on dimensionality of mental states, where boundaries between ‘health’ and ‘psychopathology’ are overlapping (Akiskal et al., 2000; Angst et al., 2010). In this view, Kirkland et al. (2015) showed that the boundaries between personality aspects of extraversion and neuroticism and happiness and hypomania were small, and above all the resolution between well-being and dysfunction was overlapped. Third, such overlapping boundaries are particularly evident when results from non-clinical samples are reported; these results suggest that among psychopathologically inconspicuous samples of children, adolescents, and adults, signs of psychopathology might emerge in regard of frequency, intensity, duration, and direction.

More specifically, the dimensionality of increased mood has gained particular attention. Holtmann et al. (2009) showed among a sample of 294 non-clinical adolescents (mean age: 17.3 years; 47.6% females) that ‘affective storms’ such as behavioral dyscontrol, agitation, increased mood, mood swings, and interactional issues were frequent. Likewise, in a former study, we showed that non-clinical adolescents during an early stage of romantic love reported hypomanic features comparable to a bipolar II diagnosis, while higher scores of hypomania might also reflect short-term stages and mood swings within a psychopathologically inconspicuous state of psychosocial development (Brand et al., 2010). Further, higher hypomania scores were associated with favorable traits of romantic love among adolescents (Bajoghli et al., 2013).

Further studies showed that higher scores of hypomania were associated with higher scores of self-efficacy, exploration behavior, pain tolerance, and coping, along with higher physical activity (Brand et al., 2011), more unstable sleep-wake patterns (Bae et al., 2014), and higher scores of creativity (Zabelina et al., 2014). Importantly, Zabelina et al. (2014) underscored the dimensionality of signs of psychopathology, as opposed to categorical entities. In this view, Gamma et al. (2008) identified 23 ‘pure hypomanics’ among a larger sample of 4547 participants of a longitudinal study. Those pure hypomanics were characterized by physical and social overactivity, elevated and irritable mood, as well as increases in extraversion, sexual interest, and risk-taking behaviors. On the flip side, they also had higher monthly incomes and were more often married than controls. Gamma et al. (2008) further reported that pure hypomanics’ subjective distress due to hypomanic symptoms was virtually absent, and quality of life and treatment rates for mood and anxiety were not different from controls (although sleep disturbances and substance abuse were descriptively more frequent). To summarize, from the study of Gamma et al. (2008) we learned that people with hypomania traits report both favorable (physical and social overactivity, higher incomes, married; no distress, equal quality of life and equal treatment of mood and anxiety disorders) and unfavorable (irritable mood, risk-taking, sleep disturbances, substance abuse) characteristics.

While there is extant literature as regards the associations between dimensions of increased scores of hypomania and romantic love states, increased sleep, and lower scores of depression and anxiety, surprisingly and to the best of our knowledge, only one study focused on the association between the dimensionality of hypomania and physical activity, while the association between hypomania scores and mental toughness is fully unexplored so far. However, we claim that focusing on both physical activity and mental toughness is justified for the following reasons: As regards physical activity, there is evidence that inactivity has become the new ‘smoking’; in other words, inactivity has become the main factor for the emergence of non-communicable diseases. Further, reviews and meta-analyses (Rosenbaum et al., 2014; Schuch et al., 2016) showed that low rates of physical activity are observed among patients with a broad variety of psychiatric disorders. In this regard, to the best of our knowledge, an increased rate of physical activity is observed in only two psychiatric disorders, that are in ADHD and in states of mania/hypomania. Here, we focus on hypomania. The first aim of the present study was therefore to associate dimensions of hypomania with self-reported rates of physical activity.

Mental toughness is a psychological concept to describe a person’s ability to cope with difficulties and to achieve self-defined aims (Clough et al., 2002). Specifically, mental toughness is an umbrella term consisting of four main dimensions (Control, Confidence, Challenge, Commitment; with subscales for Control (Control life and Control emotions) and Confidence (Confidence self and Confidence interpersonal). These dimensions describe a person’s ability to manage and control her/his life (Control; life) and emotions (Control: emotion), to have confidence in her/his own (Confidence: self) and in other people (Confidence: others), to cope with unexpected changes in life and to regard them as challenges (Challenge), and to stay focused on self-determined aims (Commitment). Initially, the concept of mental toughness (MT) was studied exclusively among elite athletes (Clough et al., 2002); now, MT has also been extensively studied among non-elite athletes such as young (Brand et al., 2016) and middle-aged adolescents (Gerber et al., 2012), healthy young adults, patients at onset of multiple sclerosis (Sadeghi Bahmani et al., 2016a), or among lower, middle, senior managers, and clerical/administrative workers in early, middle, and late adulthood (Perry et al., 2013). Higher mental toughness scores were also associated with more favorable objective sleep patterns (Brand et al., 2014a), higher coping skills (Gerber et al., 2013a,b), and higher physical activity (Brand et al., 2014a, 2016). Further, while Sadeghi Bahmani et al. (2016b) could show that higher scores of prosocial behavior and lower scores of internalizing and externalizing problems at the age of 5 years predicted higher mental toughness scores at the age of 14 years, Sabouri et al. (2016) were able to show also some ‘dark’ dimensions of mental toughness: specifically, they showed that increased mental toughness scores were also associated with dimensions of the so-called Dark Triad (along with higher physical activity scores). Briefly, Sabouri et al. (2016) showed among a sample of young adults that higher mental toughness scores were associated with higher traits of Machiavellianism, narcissism, and psychopathy, along with increased physical activity, suggesting that dimensions of mental toughness were also associated with less socially favorable and accepted behavior. To date, we know of no research, which has focused on the relation between dimensions of hypomania and mental toughness. The second aim of the present study was therefore to answer to the following research question: Are dimensions of hypomania associated with mental toughness traits?

We hold that shedding some more light on the relationship between dimensions of hypomania, physical activity and dimensions of mental toughness is important, as a deeper understanding may serve to underpin the analysis of certain facets of human behavior. Further, previous research (Holtmann et al., 2009; Brand et al., 2010) investigated dimensions of hypomania in relation to romantic love states, or in relation to symptoms of depression (Wada et al., 2013), and sleep (Brand et al., 2011). Further, Gamma et al. (2008) showed that ‘pure’ hypomanics were both socially and physically active, earning more money, prevalently married, and with equal scores of quality of life, mood and anxiety as non-hypomanics. However, as regards physical activity, to our knowledge, only one study showed that increased scores of hypomania, specifically bright-side hypomania, were associated to increased scores of physical activity (Brand et al., 2011). Thus, the present study aimed at filling these gaps of research.

The following three hypotheses and two research questions were formulated. First, following a previous study (Brand et al., 2011), we expected that higher hypomania scores would be associated with increased scores of physical activity. Second, following others (Brand et al., 2007, 2011) we hypothesized that increased hypomania scores would be associated with more favorable sleep quality, and, as third hypothesis, with lower depression scores. Next, we treated as exploratory the research questions addressing the extent to which hypomania scores would be associated with mental toughness, and which of the assessed dimensions (mental toughness traits, physical activity, sleep complaints, symptoms of depression) might best predict hypomania.

## Materials and Methods

### Procedure

Students of the Hamadan University of Medical Sciences (HUMS, Hamadan, Iran) were approached to participate in the present questionnaire-based study on the relationship between hypomania, mental toughness, physical activity, depression, and sleep. All participants were fully informed about the study aims, the confidential nature of the data gathering, and the secure data protection. Afterward, all participants signed the written informed consent. Next, clinical psychologists performed a brief psychiatric interview (Mini International Neuropsychiatric Interview; Sheehan et al., 1998) to ensure that only participants without severe mental health issues such as psychosis, suicidal behavior, or substance use disorders (opium, cannabis, alcohol, amphetamines, methamphetamines) were enrolled in the study. The University Review Board of the Hamadan University of Medical Sciences approved the study, which was conducted in accordance with the rules laid down in the Declaration of Helsinki and later amendments.

### Sample

During the spring semester 2016, a total of 263 students of the Hamadan University of Medical Sciences (HUMS; Hamadan, Iran) were approached, and 206 (80.5%; mean age: *M* = 21.3 years; age range: 18–24 years; 57.3% males) participated in the study; 15 were excluded due to psychiatric issues (substance use; severe depressive disorder), and 42 declined to participate in. No age difference between male and female participants was observed (*t* < 1, *p* > 0.4).

### Tools

#### Hypomania Check List 32 (HCL-32; Angst et al., 2005)

To assess hypomania, we employed the Hypomania Check List 32 (Angst et al., 2005), an internationally validated self-rating questionnaire to assess hypomania (Holtmann et al., 2009; Soares et al., 2010; Meyer et al., 2014), for which also the Farsi version has been psychometrically validated (Haghighi et al., 2011), and for which the transcultural approach has been acknowledged (Angst et al., 2010; Gamma et al., 2013). In the present study and in accordance to the host of previous studies with the HCL-32, we focused on calculating the 32 key – items of the questionnaire, as to the best of our knowledge, no reasonable calculation algorithms have been proposed so far for the remaining items. Typical items are: “I need less sleep,” “I enjoy my work more,” “I spend more/too much money,” “I’m less shy or inhibited,” “My thoughts jump from one topic to another,” or “I get into more quarrels.” Answers are 0 = no, or 1 = yes, with higher sum scores reflecting a higher self-rated hypomania (Cronbach’s α = 0.89).

#### Mental Toughness

Participants were asked to fill out the Mental Toughness Questionnaire 48 (henceforth MTQ48; Clough et al., 2002; Perry et al., 2013; Farsi version: Sabouri et al., 2016), which measures overall MT and its four main components or six subcomponents: challenge (e.g., “Challenges usually bring out the best in me”), commitment (e.g., “I don’t usually give up under pressure”), emotional control (e.g., “Even when under considerable pressure I usually remain calm”), life control (e.g., “I generally feel in control”), interpersonal confidence (e.g., “I usually take charge of a situation when I feel it is appropriate”), and confidence in ability (e.g., “I am generally confident in my own abilities”). Answers on the MTQ48 were given on 5-point Likert-type scales ranging from 1 = “strongly disagree” to 5 = “strongly agree.” Items were summed to obtain overall and subscale scores, with higher scores reflecting greater MT and MT traits (Cronbach’s α = 0.90).

#### Symptoms of Depression; Beck Depression Inventory (BDI; Beck et al., 1961)

Participants completed the Beck Depression Inventory (Beck et al., 1961; psychometric properties of the Farsi version: Ghassemzadeh et al., 2005), which samples self-reported symptoms of depression. The questionnaire consists of 21 items and asks about different dimensions such as depressive mood, loss of appetite, sleep disorders, suicidality and similar. Each question has a set of at least four possible responses, ranging in intensity; e.g., ‘sadness’: 0 = ‘I do not feel sad’; 1 = ‘I feel sad’; 2 = ‘I am sad all the time and I can’t snap out of it’; 3 = ‘I am so sad or unhappy that I can’t stand it,’ and with higher scores reflecting greater severity of depressive symptoms (Cronbach’s α = 0.88).

#### Sleep Disturbances

To assess sleep disturbances, the Insomnia Severity Index (ISI) was applied (Bastien et al., 2001). The ISI is a seven-item screening measure for insomnia and an outcome measure for use in treatment research. The items, answered on 5-point rating scales (0 = not at all, 4 = very much), refer in part to the Diagnostic and Statistical Manual of Mental Disorders (DSM-5) criteria for insomnia by measuring difficulty in falling asleep, difficulties remaining asleep, early morning awakenings, increased daytime sleepiness, impaired daytime performance, low satisfaction with sleep, and worrying about sleep. The higher the overall score, the more the respondent is assumed to suffer from insomnia (Cronbach’s α = 0.91).

#### Physical Activity

To obtain information on physical activity, the short version of the International Physical Activity Questionnaire (IPAQ) was used. This questionnaire was developed by a working group initiated by the World Health Organization (WHO) and the Centers for Disease Control and Prevention (CDC). The purpose of the questionnaire is to provide an estimate of a weekly physical activity time. Further, results from twelve countries demonstrate that the IPAQ has comparable reliability and validity to other self-report measures of physical activity (Booth et al., 2003). The short (self-administered, seven item), last-week version of the IPAQ was administered, which covers the time spent being physically active over the last 7 days. Minutes of sitting, walking, moderate-intensity (walking not included), and vigorous-intensity activities were computed for the last week. The IPAQ questionnaires (short and long versions), including definitions of moderate and vigorous activity, are available at www.ipaq.ki.se. However, in the present study, we collapsed the data to a composite score; the reason was that scores of vigorous-intensity activities were rarely reported and that preliminary calculations showed that differences between walking and moderate-intensity activities were very small.

### Statistical Analysis

Preliminary calculations: First, we performed a series of t-tests to compare data between male and female participants; all *t*’s were < 1.0, *p*’s > 0.1; thus, gender was not introduced as a possible confounder. Next, we performed a series of non-linear calculations between hypomania scores and scores of mental toughness, physical activity, and symptoms of depression, and the introspection showed no non-linear associations. Thus, only linear correlations were performed.

First, a series of Pearson’s correlations were performed between scores of hypomania and mental toughness, symptoms of depression, sleep complaints and physical activity. Next, to predict hypomania scores, a multiple regression analysis was performed with mental toughness traits, depression, sleep and physical activity as predictors. The nominal level of significance was set at α ≤ 0.05. All computations were performed with SPSS^®^ 24.00 (IBM Corporation, Armonk NY, United States) for Apple Mac^®^.

## Results

**Table 1** reports all correlational computations and the descriptive statistics (means, standard deviations) for the scores of hypomania and mental toughness (MTQ48), symptoms of depression (BDI), sleep complaints (ISI), and physical activity (PA).

**Table 1:** Descriptive overview and correlation coefficients between hypomania scores and mental toughness scores, symptoms of depression, sleep complaints, and physical activity.

	Descriptive statistics	
Dimensions	Hypomania	*M* (*SD*)
Mental toughness		
Challenge	0.40^∗∗^	26.90 (4.10)
Commitment	0.31^∗∗^	38.09 (5.73)
Control	0.35^∗∗^	44.73 (5.78)
Control life	0.25^∗∗^	23.86 (3.51)
Control emotions	0.30^∗∗^	20.88 (3.98)
Confidence	0.32^∗∗^	51.18 (7.78)
Confidence self	0.32^∗∗^	29.96 (5.38)
Confidence interpersonal	0.31^∗∗^	21.20 (3.74)
Mental toughness overall score	0.42^∗∗^	160.86 (19.99)
Sleep complaints (ISI)	–0.11^∗^	9.60 (5.59)
Symptoms of depression (BDI)	–0.23^∗∗^	11.74 (8.85)
Physical activity (IPAQ) (min/week)	0.17^∗∗^	2657.82 (2749.80)
Descriptive statistics *M* (*SD*)	18.67 (5.04)	

*ISI, Insomnia Severity Index; BDI, Beck Depression Inventory; IPAQ, International Physical Activity Questionnaire. ^∗^*p* < 0.05; ^∗∗^*p* < 0.01.*

Higher scores in hypomania were associated with higher scores in mental toughness, and more specifically with the overall score and with all subscales.

Higher scores in hypomania were associated with decreased sleep disturbances (ISI), and symptoms of depression (BDI), but with higher physical activity scores (PA).

### Predicting Hypomania Scores

To predict hypomania scores, a multiple regression analysis was performed with hypomania scores as dependent variable, and MT traits, sleep disturbances, symptoms of depression, and physical activity as predictors. Higher hypomania scores (*R* = 0.497; *R*^2^ = 0.243, Durbin–Watson coefficient = 1.76) were predicted by higher scores of Challenge (regression coefficient = 0.384, standard error = 0.097, β = 0.395, *t* = 3.944, *p* = 0.006) and Control (regression coefficient = 0.176, standard error = 0.068, β = 0.208, *t* = 2.610, *p* = 0.010), and higher physical activity scores (regression coefficient = 0.130, standard error = 0.048, β = 0.140, *t* = 2.036, *p* = 0.043), while all other predictors were excluded from the equation (βs < 0.1, *p*s > 0.1; Commitment, Confidence, sleep disturbances, symptoms of depression; intercept: regression coefficient = -0.268, standard error = 2.86, *t* = -0.094, *p* = 0.925).

## Discussion

The key findings of the present study were that among a healthy sample of university students, higher hypomania scores were associated with higher scores of all traits of mental toughness and physical activity, along with lower scores of symptoms of depression and sleep complaints. Further, higher scores of Challenge and Control and physical activity predicted higher hypomania scores. The present study adds to the literature in an important way as we showed a favorable trait of hypomania, which was tightly related to increased dimensions of mental toughness and physical activity.

Three hypotheses and two research questions were formulated and each of these is now considered in turn.

With the first hypothesis we expected that higher hypomania scores would be associated with higher physical activity scores, and data did confirm this. Thus, the data are in accord with a previous study (Brand et al., 2011). Here, we expand upon previous research in that we observed a linear and positive association between hypomania and subjective duration of physical activity. Thus, following this pattern of results, people from a non-clinical sample in a higher mood are also more physically active (and vice versa).

With the second hypothesis we expected that increased hypomania scores would be associated with fewer sleep disturbances, and again, data did confirm this. Accordingly, the present data are in accord with previous results (Brand et al., 2007, 2011). The novelty, however, of the data is that we did not focus on people during an early stage of romantic love, but on a non-clinical sample of young adults. Further, the present pattern of results was in accord with recent data on the association between mood states and sleep quality among a larger non-clinical sample of adults: Oniszczenko et al. (2017) showed that a hyperthymic temperament was negatively correlated with insomnia symptoms. Further, most importantly, we solely focused on sleep complaints, that is, on sleep quality, which has been shown to be more important for subjective well-being (Pilcher et al., 1997) and injury-related behavior (Edmonds and Vinson, 2007) than sleep quantity data such as sleep duration. Collectively, the pattern of results is in accord with the notion that sleep need decreases during increased mood states, though, as the present data suggest, such an association is not related to a deterioration of subjectively experienced sleep quality.

With the third hypothesis we expected that higher hypomania scores would be associated with lower depression scores, and data did confirm this. Thus, the pattern of results suggests that hypomania and depression appear to be considered opposite poles of a continuum of mood states.

With the first exploratory question we considered the extent to which hypomania scores were associated with mental toughness, and data showed that increased hypomania scores were associated with higher MT scores of all traits. To our knowledge, this is the very first study to show such an association. We hold that the present data are of importance, as we observe a growing interest in the exploration of mental toughness because it has the power to explain a broad variety of behavior such as more favorable objective sleep, improved coping strategies (Gerber et al., 2013a,b, 2015), and is applicable across a range of different samples, such as patients with MS (Sadeghi Bahmani et al., 2016a), lower, middle, and senior managers, and clerical/administrative workers in early, middle, and late adulthood (Perry et al., 2013).

The present results also answer the second research question: Higher hypomania scores were predicted by higher scores of Challenge and Control, along with higher physical activity scores. We also note that *all* MT traits were positively correlated with higher hypomania scores (see **Table 1**), though the inherent statistical algorithms of multiple regression analyses are such to favor those predictors with higher correlation coefficients.

The quality of the data does not allow a deeper introspection into the underlying psychological mechanisms of why hypomania and mental toughness might be associated, though we advance the following hypotheses: typical items of the hypomania questionnaire (Angst et al., 2005; Brand et al., 2010; Haghighi et al., 2011) cluster on being both physically and mentally more active, reporting higher self-esteem, positive social interactions and risky behavior (Haghighi et al., 2011), thus, behavior also included in items of the mental toughness questionnaire (MTQ48; Clough et al., 2002; Gerber et al., 2012, 2013a,b; Brand et al., 2014a,b, 2016) such as keeping track of activities, seeking challenges, or feeling confident in oneself and in other people. Thus, we observe an overlap between hypomania traits and mental toughness traits both at item and at behavioral level. In this view, it is not surprising that those reporting higher hypomania scores (“being both physically and mentally more active”) were also those participants scoring higher in the physical activity questionnaire.

Despite the novelty of the data, several limitations warrant against an overgeneralization of the results. First, we assessed young adults, who by definition do not represent people of lower and higher age, and above all not people with mental health issues. In this view, both inclusion and exclusion criteria were such to exclude participants with psychiatric disorders. While from a scientific point of view, it would have been also important to assess such participants, we decided to exclude them, as we wanted to investigate explicitly a non-clinical sample. Second, we did not distinguish between ‘bright’ and ‘dark’ side hypomania categories, as proposed by other studies (Meyer et al., 2007; Holtmann et al., 2009; Brand et al., 2015), though we focused here on the continuum of low to high hypomania scores, and not in its dichotomous or higher order categories (Angst et al., 2010; Haghighi et al., 2011; Zabelina et al., 2014; Kirkland et al., 2015). Third, the cross-sectional nature of the study precludes the determination of causal relationships, which could only be solved with a longitudinal study design. Fourth, the present pattern might have emerged due to further latent and unassessed variables, which biased two or more variables in the same or opposite directions. Fifth, we relied on a subjective estimation of physical activity, while activity trackers such as actigraphs would have been a more accurate and reliable source of physical activity. Sixth, we did not further assess further items of the HCL-32 such as “Negative consequences” or “Reactions of others” (see Angst et al., 2005; Meyer et al., 2007), though, to our knowledge, no thorough calculation algorithms to integrate these data have been proposed so far. Seventh and last, the selection and the quality of the tools did not allow to distinguishing between hyperthymic temperament, high affective intensity, and hypomania traits. While we preferred to focusing on hypomania traits, future studies might investigate, if the present pattern of results might be observable also for people with a hyperthymic temperament or a high affective intensity.

## Conclusion

Among a sample of young adults, higher hypomania scores were associated with higher scores of mental toughness and physical activity. The results add to the current literature in showing that traits of hypomania are associated with favorable traits such as mental toughness and increased physical activity.

## Author Contributions

Study design: LJ, AR, MH, MA, DSB, AS, SS, HB, MG, and EH-T and SB. Data gathering: LJ, AR, MH, MA, DSB, AS, SS, and HB. Data analysis: AR, DSB, MG, EH-T, and SB. Interpretation of the data: LJ, AR, MH, MA, DSB, AS, SS, HB, MG, EH-T, and SB. Writing the first draft: AR, MA, DSB, MG, EH-T and SB. Integration of the authors’ comments: AR, MA, DSB, MG, EH-T, and SB. Final manuscript: LJ, AR, MH, MA, DSB, AS, SS, HB, MG, EH-T, and SB.

## Conflict of Interest Statement

The authors declare that the research was conducted in the absence of any commercial or financial relationships that could be construed as a potential conflict of interest.
